# Impact of a Light Volleyball Intervention Program on Improving Physical Attributes of Older Adults in Hong Kong: Preliminary Study of a Randomized Controlled Trial

**DOI:** 10.2196/62886

**Published:** 2025-05-28

**Authors:** Ka Man Leung, Yuchen Shi

**Affiliations:** 1 Department of Health and Physical Education, Faculty of Liberal Arts and Social Science, Education University of Hong Kong, Tai Po, China (Hong Kong); 2 School of Liberal Arts, Macau University of Science and Technology, Avenida Wai Long, Taipa, Macau, China, 853 87967830

**Keywords:** adapted physical activity, older adults, gerontology, geriatrics, older, aging, randomized controlled trial, volleyball, light volleyball, intervention, sports, physical activity, exercise, physical attributes, RCT, controlled trial

## Abstract

**Background:**

Physical inactivity, which increases the risk of chronic diseases in older adults, is prevalent among older adults in Hong Kong. To address this problem, the Hong Kong government has been proactively promoting active aging.

**Objective:**

Following the World Health Organization’s strategy to prevent chronic diseases in older adults and aligning with the global goal of active aging, this study evaluated the effects of a 16-week light volleyball (LVB) intervention program on the physical health of older adults in Hong Kong.

**Methods:**

A total of 276 participants aged ≥60 years were recruited and randomly assigned to 1 of 3 groups: an LVB intervention group, a Taichi control group (ie, with light physical activity), and a control group. Tests on components of fitness were conducted before and after the intervention.

**Results:**

Participants from the LVB intervention group exhibited significant improvements in lower body strength (*F*_2,272_= 7.23, *P*=.001, η^2^=.05), agility (*F*_2,272_= 6.05, *P*=.003, η^2^=.043), and dynamic balance (*F*_2,272_= 9.41, *P*=.001, η^2^=.065) when compared with those from the Taichi active control group and control group.

**Conclusions:**

To promote active aging among older adults in Hong Kong, the findings of this preliminary study, along with forthcoming follow-up tests, will provide health specialists and practitioners with valuable insights regarding the health benefits of the LVB community program for older adults.

## Introduction

Given the low birth rate and increasing life expectancy in Hong Kong, the trend toward an aging population is expected to persist in the coming decades in this region. The number of individuals aged ≥65 years is projected to rise substantially, reaching 1.89 million by 2019 and 30.5 million by 2069 [1]. This continual demographic shift highlights several societal challenges, including a decline in the working population and an increased burden on Hong Kong’s social welfare and health care systems. With an expanding share of older adults in the population, public health expenditure in Hong Kong is forecasted to increase by 394%, from US $4.9 billion in 2004 to US $24.4 billion in 2033, accounting for 5.5% of Hong Kong’s total GDP in 2033 [2]. The simultaneous decrease in the working population and increase in social welfare expenditures for the aging population are expected to create severe financial strain for the Hong Kong government in the coming decades.

Although the benefits of physical activity have been well-documented [3], approximately 40% of older adults aged 60 years or older in Hong Kong engage in insufficient physical activity [4]. More than 50% of older adults in Hong Kong have overweight or obesity [5], and 75% have one or more chronic illnesses [6]. Additionally, older adults in Hong Kong spend an average of 7.5 hours sitting and 6.7 hours lying down daily [7]. Spiteri et al [8] identified various barriers to physical activity for older adults, including perceptions of negative consequences (ie, pain, risk of injury, and fear of falling), and a lack of knowledge and skills. Similar barriers to physical activity have been reported among older adults in Hong Kong. To address these challenges, promoting active aging through physical activity is essential [9]. The World Health Organization (WHO) [10] introduced the “Active Ageing: A Policy Framework,” warning that failure to address the aging population crisis could lead to the collapse of health care and social welfare systems. The Hong Kong government interprets active aging as achieving full physical, social, and mental well-being [6]. Since 2001, it has developed a healthy aging report outlining strategies to promote active aging, including physical activity initiatives. For instance, the Hong Kong government installed 150 new fitness equipment sets for older adults across Hong Kong between 2017 and 2018 to encourage active aging [11].

Meta-analyses have demonstrated that physical activity interventions have a significant effect on improving balance, reducing falls [12], and enhancing physical function [13] in older adults. Both supervised resistance and aerobic physical activity interventions have demonstrated positive effects on physical function outcomes. Future research directions include (1) increasing the number of physical activity interventions conducted in Asia, (2) aligning intervention programs with the WHO’s recommendation of 150 minutes of physical activity per week, (3) using more rigorous randomized controlled trial designs, and (4) implementing supervised physical activity interventions with larger sample sizes in Asia [14,15]. Recent reviews have emphasized the need to examine the effects of sports interventions on older adults’ health, particularly among Chinese older adults. Wong et al [16] reviewed 371 studies from the past 15 years on the effects of physical activity on older adults’ physical health, and they recommended exploring the health benefits of newly emerging sports, such as light volleyball, in future research [16,17]. Franco et al [17] qualitatively reviewed the literature on older adults’ perceptions of physical activity participation, suggesting that older adults prefer physical activity programs that are (1) professionally instructed, (2) group-based and conducive for peer interaction, and (3) highly accessible, with affordability being a key factor influencing the willingness to participate [17]. Similar findings were reported by Van Dyck et al [18], who highlighted older adults’ preferences for innovative physical activity activities, such as aqua fitness or light volleyball, over traditional options, such as walking and cycling, in intervention programs.

Light volleyball (LVB) is a newly adapted physical activity derived from traditional volleyball. Compared with traditional volleyball, LVB uses balls with a larger circumference (80‐83 cm vs 65‐67 cm) and lighter weight (150g vs 250g), allowing the balls to travel at a slower speed. These features make LVB more accessible, particularly for individuals with reduced physical capacity due to age-related decline (eg, older adults). Additionally, the LVB court is smaller (similar in size to a badminton court), and the net is set lower (1.8 m), further reducing the physical demands on participants. Studies from China have reported physical health benefits associated with regular LVB practice; however, most of these studies did not have standardized fitness measurements and control groups. In 2020, the first author and her team conducted a quasi-experimental intervention to examine the effects of LVB on the physical and psychological health attributes of 78 older adults aged ≥60 years. The results indicated significant improvements in physical (ie, lower and upper body muscle strength, agility, balance, and aerobic endurance) and psychological (ie, physical activity enjoyment) attributes among participants in the LVB group when compared with the control group [19]. Furthermore, improvements in upper body muscle strength, aerobic endurance, and physical activity enjoyment were significantly more pronounced in the LVB group than in the active control group, which participated in Rouliqiu. That study highlighted the effectiveness of LVB intervention programs in enhancing both physical and psychological health attributes in older adults. Despite these promising findings, that study had notable limitations. First, its sample size was small, with only 62 participants completing the screening tests, pre- and postintervention functional tests, and data analysis. Second, participants were not randomly assigned to groups, which could have introduced selection bias.

Building on the positive results of the LVB pilot study and the prioritization of resource allocation for promoting active aging in Hong Kong, the first author and her team secured Research Impact funding amounting to US $0.95 million for further research in this area. This funding supported an investigation into the effectiveness of an LVB intervention on the physical and psychological health attributes of older adults in Hong Kong using both quantitative and qualitative methods and examining a larger sample size of approximately 300 participants. In this study, we present the preliminary results of this LVB intervention in our quantitative arm using the results from the pretest and posttest. In the current study, Taichi was selected as the intervention for the active control group because both Taichi and LVB are whole-body exercises originating from China and suitable for older adults [20,21]. Compared with the team-based LVB, Taichi is an individual exercise, which may lead to differing effects on older adults’ health and quality of life [22]. Prior research suggests that older adults with greater social support are more likely to continue exercising regularly [23,24]. Community-based group physical activity interventions with increased social support have also been associated with greater beneficial effects and program adherence. Additionally, Taichi is a popular activity among Chinese older adults [25]. With LVB gaining traction in Hong Kong, Taichi serves as a relevant comparison, providing valuable insights into physical activity promotion among Hong Kong’s older adult population. This study aimed to assess the effects of a 16-week LVB intervention compared with Taichi and a control group on two key outcomes in Chinese older adults aged ≥65 years: (1) functional fitness and (2) balance. The hypothesis was that both LVB and Taichi would result in significant and comparable improvements in these physical health attributes relative to the control group.

## Methods

### Study Design

A randomized controlled trial design was used to assess the effects of the LVB intervention on participants’ physical health outcomes. Following the CONSORT (Consolidated Standards of Reporting Trials) guidelines (Checklist 1) [26], detailed information about the intervention can be found in our previously published study protocol (trial registration number ChiCTR1900026657) [19]. The LVB group was compared against an active control group (ie, Taichi) and a control group. Participants were randomly assigned into LVB group, Taichi group, and control group in 1:1:1 ratio.

### Study Intervention

The intervention program was conducted from mid-2021 to early-2023, with data collection performed at pretest (immediately before the intervention) and posttest (immediately after the intervention). The preliminary results presented here are based on pretest and posttest data only. Participants in the LVB and Taichi groups attended a 16-week training program consisting of two 90-minute sessions per week following the pretest. This intervention duration aligns with the guidelines of the Centers for Medicare and Medicaid Services of the US Department of Health and Human Services, which suggest that the health benefits of physical activity for older adults can be observed within 1 to 3 months after the start of a program [27]. In contrast, the control group was instructed to continue their usual daily activities while participating in monthly social gatherings (eg, health workshops) to control for psychosocial effects.

### Participants

Participants were eligible for recruitment if they (1) were aged ≥60 years, (2) lived independently, (3) had no cognitive impairment, (4) had not participated in physical activity programs for 2 consecutive years prior to the program, and (5) had passing scores on the Abbreviated Mental Test (AMT) and Timed-up-and-go (TUG) test [28,29]. Specifically, individuals had to obtain a score of at least 6 out of 10 on the AMT to demonstrate sufficient cognitive capability and complete the TUG test within 20 seconds. Participants were ineligible for recruitment if they had steady hypertension (≥160/90 mmHg), arthritis, or neurological disorders.

### Recruitment and Procedures

Participants were recruited through informational sessions conducted by the research team and advertisements placed in local neighborhood elderly centers. All participants were informed about the confidentiality of their personal data collected for the study and were assured they could withdraw from the program at any time. The research team sought consent from each participant before collecting data and proceeded immediately to conduct the AMT and TUG test to screen unqualified participants. After participants passed the screening tests, they completed the following steps: (1) questionnaires, (2) sociodemographic questions, (c) measurements of weight and body fat percentage using the Tanita machine (TBF-410GS), and (d) a functional fitness test based on the work of Leung et al [19,20]. After the pretest, an independent researcher used a computer-generated random number system to assign participants to the intervention conditions. Data collection and entry personnel were blinded to the group assignments of participants. The intervention program commenced the following week in sports complexes at community centers or community halls in Hong Kong and lasted for 16 weeks (approximately 3.5 months). The research team arranged the posttest for participants within 7 days after they completed the intervention program. All participants received a supermarket cash voucher worth US$ 12.84 as an incentive for their participation.

### Ethical Considerations

The study was reviewed and approved by the Education University of Hong Kong’s Research Ethics Committee (approval number E2022-2023-0013).

### Measures

#### Functional Fitness

The research team used the Senior Fitness Test Manual to assess the physical attributes of older adults [30]. The test comprises 6 items: the chair stand test (lower body strength), arm curl test (upper body strength), chair sit-and-reach test (lower body ﬂexibility), back-scratch test (upper body ﬂexibility), 8-foot up-and-go test (agility and balance), and 2-minute step test (aerobic endurance). These have been demonstrated to be reliable, with intraclass correlation coefficients ranging between .80 and .98 in participant trials. Their validity has been supported through content, criterion-related analyses, and construct validation, including comparisons of senior fitness test scores with other established measures, such as treadmill VO2 testing [31]. Higher scores in the chair stand test (repetitions), arm curl test (repetitions), chair sit-and-reach test (cm), back-scratch test (cm), and 2-minute step test (repetitions) indicate higher levels of lower and upper body strength, flexibility, and aerobic endurance; the converse is the case for the 8-foot up-and-go test (seconds). For the flexibility assessments, negative scores reflect an inability to reach the toes during the chair sit-and-reach test or to make hand contact in the back-scratch test.

#### Balance Test

The Balance System SD (BBS-SD, 950‐441 model) was used to measure the dynamic balance of older adults in the current study. Participants were asked to stand upright at the center of the platform while observing a screen situated 30 cm (approximately 11.1 in) in front of them. They completed three 20-second trials with 10-second breaks in between. The results of 3 trials were collected, and the mean values were recorded by the research team. The findings of previous studies support the reliability and validity of this balance test [32,33]. The measurement index and overall stability index generated by the Biodex balance system were included in the current study. These indices measured the participants’ dynamic balance, specifically assessing their balance fluctuations across multiple axes. Higher values indicated greater deviations and poorer balance control.

### Data Analysis

Data were analyzed using SPSS 27.0 software (IBM Corp). Descriptive statistics, specifically mean and SD values for continuous variables and frequencies and percentages for categorical variables, were used to describe the data. Preliminary checks were conducted to ensure that the assumptions of normality and homogeneity of variance were met. One-way ANOVA was performed to evaluate baseline differences between the groups. To examine the effect of the 16-week intervention program on physical attributes, a series of analysis of covariance tests were conducted, comparing the 3 groups (LVB, Taichi, and control groups) at 2 time points (pre- and posttest). Body mass was used as a covariate because of its correlation with the outcome measures [34]. Partial eta squared (η^2^), *P* values, and contrast *t*-statistics were calculated. Planned contrasts were used to analyze the differences between the LVB and control groups, and between the LVB and Taichi groups. Cohen *d* was calculated as a measure of effect size, with thresholds of 0.1 for a small effect, 0.3 for a medium effect, and 0.5 for a large effect [35]. Statistical significance was set at *P*<.05.

## Results

### Overview

**Figure 1.**
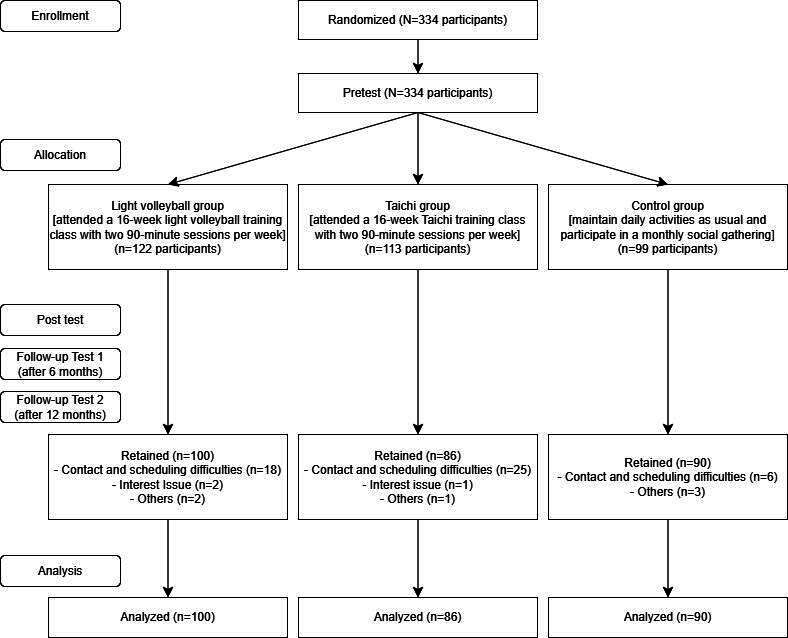
Recruitment statistics.

Figure 1 presents the recruitment process for the current study. At the start of the intervention, 334 older adults from 7 elderly centers were enrolled in the program and completed the pretest. With simple randomization, there were 122 older adults in LVB group, 113 in Taichi group, and 99 in control group. A total of 3 participants (2 from the LVB group and 1 from the Taichi group) withdrew from the program because of personal reasons, and 7 others (2 from the LVB group, 1 from the Taichi group, and 3 from the control group) withdrew without providing any reason. Among the remaining 318 participants, 42 did not complete the posttest for various personal reasons (eg, illness, Lunar New Year gatherings, and concerns about social distancing during the late COVID-19 pandemic). This sample size met our calculated sample size for this intervention (with effect size of 0.5 [Cohen *d*]) in order to achieve a power of 80% at a significance level of 5% [19]. The retention rate was 82.6%, slightly higher than the expected 80%. Ultimately, 276 participants (LVB group, n=100; Taichi group, n=86; control group, n=90) who completed both the pre- and posttest were included in the final data analysis.

Table 1 presents the sociodemographic characteristics of the participants. Approximately 56% (156/276) of the participants were aged ≤70 years, and only 8% (22/276) were aged ≥80 years. The majority of the participants were female (229/276, 83%) and retired (217/276, 78.6%). Approximately 57.3% (158/276) had attained secondary education or higher. Approximately half (161/276, 58.3%) reported their financial status as average. The average BMI was 24 (SD 3.6) kg/m^2^. The groups did not significantly differ with each other at the pretest with respect to all variables (*P*>.05).

**Table 1.** Sociodemographic characteristics of participants.

Characteristics	LVB^a^ (n=100)	TC^b^ (n=86)	Control^c^ (n=90)
Age (years), n (%)			
60‐64	30 (30)	14 (16.3)	10 (11.1)
65‐69	35 (35)	34 (39.5)	33 (36.7)
70‐74	24 (24)	26 (30.2)	17 (18.9)
75‐79	8 (8)	8 (9.3)	15 (16.7)
80 or above	3 (3)	4 (4.7)	15 (16.7)
Sex, n (%)			
Male	17 (17)	18 (20.9)	12 (13.3)
Female	83 (83)	68 (79.1)	78 (86.7)
Occupation, n (%)			
Full-time job	0 (0)	0 (0)	1 (1.1)
Housewife	16 (16)	11 (12.8)	21 (23.3)
Retired	79 (79)	72 (83.7)	66 (73.3)
Part-time job or others	5 (5)	3 (3.5)	2 (2.2)
Education level, n (%)			
No education	5 (5)	4 (4.7)	21 (23.3)
Primary education	35 (35)	23 (26.7)	30 (33.3)
Secondary education	40 (40)	41 (47.7)	29 (32.2)
Tertiary education	20 (20%)	18 (20.9%)	10 (11.1%)
Perceived financial status, n (%)			
Low	19 (19)	18 (20.9)	18 (20)
Below average	21 (21)	17 (19.8)	10 (11.1)
Average	53 (53)	50 (58.1)	58 (64.4)
Above average	3 (3)	1 (1.2)	3 (3.3)
Higher	4 (4)	0 (0)	1 (1.1)
House nature, n (%)			
Bought	46 (46)	33 (38.4)	32 (35.6)
Rent	54 (54)	53 (61.6)	58 (64.4)
BMI (kg/m^2^), mean (SD)	24.1 (3.7)	23.5 (3.7)	24.2 (3.4)

a LVB: light volleyball.

b TC: Taichi.

c CG: control group.

### Improvement in Physical Fitness

Table 2 presents the mean and SD values of physical measures for the groups at pre- and posttest, along with the results of analysis of covariance with repeated measures, with BMI controlled for. After the intervention, the LVB group achieved statistically significant improvements in lower limb muscular endurance (chair stand test, *F*_2,272_= 7.2, *P*=.001, η^2^=.05), agility (up-and-go test, *F*_2,272_= 6.1, *P*=.003, η^2^=.04), and dynamic balance (Biodex balance test, *F*_2,272_= 9.4, *P*=.001, η^2^=.07) compared with the other groups.

Pairwise comparisons revealed that both the LVB and Taichi groups exhibited significantly improved adjusted performance in lower limb muscular endurance (chair stand test, LVB group, mean 17.6, SD 5.0; Taichi group, mean 15.3, SD 4.9, *P*=.001), agility (up-and-go test, LVB group, mean 5.6, SD 1.3; Taichi group, mean 6.2, *P*=.001), and dynamic balance (Biodex balance test, LVB group, mean 0.5, SD 0.5; Taichi group, mean 0.7, SD 0.6, *P*=.001) compared with the control group (chair stand test, mean 15.3, SD 4.9; up-and-go test, mean 6.1, SD 1.2; Biodex balance test, mean 0.7, SD 0.5). Notably, the LVB group performed significantly better on all three measures (chair stand test, up-and-go test, and Biodex balance test) at posttest compared with the Taichi group. No significant group differences were identified for the arm curl test (*F*_2,270_=2.8, *P*=.1, partial η^2^=.02), chair sit-and-reach test (*F*_2,270_=0.06, *P*=.5, partial η^2^=.005), and back scratch test (*F*_2,270_=1.3, *P*=.3, partial η^2^=.01), and 2-min step test (*F*_2,270_=1.1, *P*=.3, partial η^2^=.008).

**Table 2.** Mean and SD values for measures in groups at pre- and posttest.

Measures	LVB^a^(n=100), mean (SD)		TC^b^(n=86), mean (SD)		CG^c^(n=90), mean (SD)		*F* test (*df*)	Mean difference (95% CI)	
	Pretest	Posttest	Pretest	Posttest	Pretest	Posttest		LVB-TC	LVB-CG
Chair stand test (frequency)	16.4 (5.1)	17.6 (5.0)	15 (5.1)	15.3 (4.9)	15 (3.7)	15.3 (3.5)	7.2^d^ (2, 272)	2.4^d^(0.8 to 3.9)	2.3^d^(0.8 to 3.9)
Arm curl (frequency)	15.3(4.9)	16.0 (4.8)	14.4(5.1)	14.6 (4.9)	14.1(5.3)	14.2 (5.0)	2.8 (2, 270)	1.5(−0.2 to 3.2)	1.9^e^(0.2 to 3.5)
Chair sit-and-reach test (cm)	7.3(11.5)	7.8 (11.4)	5(13.0)	5.2 (12.6)	5.1(9.5)	4.8 (8.8)	0.7 (2, 272)	2.7(−0.8 to 6.1)	3(−0.4 to 6.4)
Back scratch (cm)	0.6(9.3)	1.0 (9.5)	0.9(8.9)	0.1 (8.7)	−2 (9.2)	−2.7 (9.4)	1.3 (2, 270)	0.9(−1.9 to 3.6)	3.7^e^(0.1 to 6.3)
Up-and-go test (s)	6 (1.5)	5.6 (1.3)	6.2(1.8)	6.2 (1.7)	6.5(1.4)	6.1 (1.2)	6.1^d^ (2, 272)	−.6^e^(−1.1 to −0.1)	−.7^d^(−1.2 to −0.2)
Step test (frequency)	92.1(18.3)	94.6 (17.6)	89.4(22.9)	90.0 (21.8)	85.3(17.6)	85.9 (16.7)	1.3 (2, 272)	4.56(−1.5 to 10.7)	8.7^e^(2.7 to 14.7)
Overall stability index (score)	0.7(0.6)	0.5 (0.5)	0.7(0.6)	0.7 (0.6)	0.8(0.6)	0.7(0.5)	9.4^d^ (2, 272)	−0.2^e^(−0.3 to −.01)	−0.2^d^(−0.4 to −0.04)

a LVB: light volleyball.

b TC: Taichi.

c CG: control group.

d *P*<.01.

e *P*<.05.

## Discussion

### Principal Findings

Given the continued aging of the population in Hong Kong and the limitations of our previous LVB pilot study, the current study aimed to investigate the effect of a 16-week LVB intervention program on physical health outcomes among older adults. Compared with the pilot study, the current study used an randomized controlled trial design and recruited 5.45 times more participants. The results indicated that the LVB intervention had a greater effect on improving lower body strength, agility, and dynamic balance in older adults when compared with both the Taichi intervention and the control group. Although significant improvements in lower body strength, agility, and dynamic balance were identified in the LVB intervention group, no significant changes in aerobic endurance and upper body strength were identified.

### Improvement in Physical Health

The current study hypothesized that the LVB intervention would lead to greater improvements in lower body strength, agility, and dynamic balance relative to the control. This expectation is supported by our pilot study in 2018, which revealed significant improvements in these physical attributes for the LVB intervention group when compared with the control group [20].

We also hypothesized that the LVB intervention would improve lower body strength, agility, and dynamic balance relative to Taichi. First, LVB shares several similarities with traditional volleyball, such as involving a considerable amount of lower body movement, which helps enhance lower body strength [36]. Second, LVB was expected to result in more significant improvements in agility and dynamic balance compared with Taichi. This is because LVB requires players to use open skills, enabling them to react and adapt to dynamic, constantly changing environments. In contrast, Taichi primarily involves closed skills, which do not require such adaptive responses to environmental changes [37]. Although there may be differences, Sheppard and Young [38] defined agility as the ability to change speed or direction in response to a stimulus, such as an environmental change. A recent systematic review comparing the effects of open-skill physical activity and closed-skill physical activity on cognitive function reported that open-skill physical activity was more effective in enhancing cognitive function than closed-skill exercises were [39]. Furthermore, Young et al [40] suggested that agility is strongly linked to cognitive functions such as decision-making ability and perception.

Additionally, a strong correlation has been observed between lower body strength and the ability to change direction (change-of-direction ability) [41]. Recent research has demonstrated a significant correlation between relative and absolute strength and agility, including change-of-direction ability and linear speed [42]. In the current study, we also identified a correlation between lower body strength and agility. These were the 2 physical attributes for which more pronounced improvements were identified in the LVB intervention group than in the Taichi group. In addition to lower body strength, balance plays a crucial role in maintaining good posture during acceleration, deceleration, and sudden changes in location or direction. Balance training has been found to be beneficial in improving the agility of volleyball players [43].

Although the participants in the LVB group exhibited greater improvements in lower body strength, agility, and dynamic balance, an unexpected finding was the minimal differences in upper body strength and aerobic endurance, 2 physical attributes that were initially hypothesized to exhibit significant improvement in the LVB group compared with the Taichi group. For upper body strength, LVB involves more frequent and vigorous arm and shoulder movements relative to Taichi, such as spiking and blocking, which were expected to result in greater upper body strength improvements compared with Taichi [44]. Similarly, the higher energy demand in volleyball was assumed to lead to greater improvements in aerobic endurance in the LVB group compared with Taichi [45]. However, despite these expectations, no significant differences in improvements were observed between the LVB and Taichi groups for these two physical attributes. Nonetheless, both the LVB and Taichi programs were demonstrated to improve upper body strength and aerobic endurance in older adults, although LVB did not significantly outperform Taichi as was hypothesized. Previous studies have indicated that both LVB and Taichi can be beneficial for improving upper body muscle strength in older adults [19,44]. Regarding aerobic capacity, although we assumed that volleyball would require higher aerobic endurance,Taichi’s breathing techniques were found to be beneficial for improving aerobic capacity in older adults, providing benefits similar to those achieved through LVB [46].

### Limitations

Although this study addressed the limitations of the previous pilot study, it still has several limitations of its own. First, these are only preliminary findings, and the scheduled follow-up evaluation in 6 or 12 months will help determine whether these advantages are seen or sustained over the longer term after the intervention. Second, the gender representation was disproportionate; the number of female participants was higher because recruitment was conducted through local elderly centers. These centers in Hong Kong have predominantly female memberships, with older women showing a greater tendency to participate in activities [47,48]. This imbalance in gender representation limits the generalizability of the findings. Furthermore, the study was conducted during the COVID-19 pandemic and the proposed intervention period was postponed from 2020‐2022 to 2021‐2023, which may have introduced variables that are not typically present in nonpandemic conditions. These factors could have affected the results of the interventions.

### Conclusions

The current study examined the effects of a 16-week LVB intervention program on the physical health of older adults in Hong Kong. The results indicated that the participants in the LVB group experienced significant improvements in lower body strength, agility, and dynamic balance compared with both the Taichi active control group and the control group. This study builds on the previous pilot by adopting an randomized controlled trial design, incorporating dynamic balance as a fitness component, increasing the sample size, and collecting data from 7 local elderly centers instead of just one.

Future studies should address the following: first, studies should aim for more balanced gender representation and include follow-up tests to monitor the long-term maintenance of physical improvements. Numerous studies have highlighted the positive effects of physical activity on aspects such as cognition and psychology in older adults, whereas the current study focused solely on physical attributes. Future research could explore the effects of LVB on cognitive and psychological outcomes to provide a more comprehensive understanding of its benefits. Furthermore, future studies should consider qualitative approaches to understand participants’ experiences and assess how LVB intervention influences various health dimensions. Given the potential limitations due to the COVID-19 pandemic, future studies may benefit from replication under more stable conditions, free from social distancing policies, to determine whether similar results can be observed. In conclusion, this large-scale study provides strong evidence supporting the physical health benefits of LVB for older adults. The results from our future qualitative studies and follow-up measures will further inform researchers and practitioners about the acceptability and appropriateness of LVB interventions for older adults.

## Supplementary material

10.2196/62886 Checklist 1 CONSORT Checklist

## Abbreviations

AMT: Abbreviated Mental Test
CONSORT: Consolidated Standards of Reporting Trials
LVB: light volleyball
TUG: Timed-Up-and-Go
WHO: World Health Organization
